# CaMKII modulates memory destabilization by regulating the interaction of theta and gamma oscillations

**DOI:** 10.3389/fncel.2025.1620588

**Published:** 2025-08-21

**Authors:** Janine I. Rossato, Maria Carolina Gonzalez, Johseph P. G. Souza, Andressa Radiske, Gênedy Apolinario, Ana Luizi Baracho, Martín Cammarota

**Affiliations:** ^1^Memory Research Laboratory, Brain Institute and Department of Physiology and Behavior, Federal University of Rio Grande do Norte, Natal, Brazil; ^2^International Institute of Neuroscience, Macaiba, Brazil

**Keywords:** memory, reconsolidation, CaMKII, theta oscillations, gamma oscillations, hippocampus, memory destabilization, object recognition memory

## Abstract

Object recognition memory (ORM) allows animals to distinguish between novel and familiar items. When reactivated during recall in the presence of a novel object, a consolidated ORM can be destabilized and linked to that generated by the novel object through reconsolidation. The CA1 region of the dorsal hippocampus contributes to ORM destabilization and reconsolidation, with mechanisms involving theta/gamma cross-frequency coupling (hPAC) and synaptic plasticity modulation. Ca^2^+/calmodulin-dependent protein kinase II (CaMKII) is vital for hippocampus-dependent memory processing and has been associated with theta activity-dependent plasticity in dorsal CA1. However, the specific role of hippocampal CaMKII in the lasting storage of reactivated ORM remains unclear, and its potential impact on memory-related oscillatory activity has not been previously investigated. To explore these questions, we employed a combination of behavioral, electrophysiological, and pharmacological approaches at various stages of ORM processing, and found that CaMKII is not necessary for ORM recall or reconsolidation but does regulate novelty-induced ORM destabilization by modulating hPAC.

## Introduction

Object recognition memory (ORM) is a form of episodic memory that enables animals to identify familiar items and distinguish them from novel ones (Broadbent et al., 2009). In rats, consolidated ORMs are durable but they can be temporarily destabilized when reactivated alongside new, relevant information during recall (Rossato et al., 2007; Winters et al., 2011). When triggered by the perception of a novel object next to a familiar one in a familiar non-aversive environment, ORM destabilization allows for the association of the memories of these objects (Gonzalez et al., 2021a). This process involves a hippocampal protein synthesis-dependent memory re-stabilization mechanism known as reconsolidation (Lee et al., 2017; Nader et al., 2000). Specifically, novelty-induced ORM destabilization requires activation of GluN2B-NMDAR in the hippocampus, induces phase-amplitude cross-frequency coupling of theta-gamma oscillations in dorsal CA1 (hPAC), and is associated with a brief period of CA3-CA1 synaptic depotentiation following memory recall. In contrast, ORM reconsolidation leads to a late increase in hippocampal synaptic efficacy, characterized by NMDAR and PKMζ-dependent up-regulation of AMPAR trafficking (Clarke et al., 2010; Rossato et al., 2019; 2023; Gonzalez et al., 2021b).

The serine/threonine protein kinase Ca2+/calmodulin-dependent protein kinase II (CaMKII) is a critical mediator of memory processing in the hippocampus (Nicoll and Schulman, 2023), where it directly interacts with GluN2B-NMDAR at the postsynaptic side of glutamatergic synapses, modulating AMPAR gating, synaptic targeting, and downstream signaling (Lisman et al., 2012) as well as plasticity in the theta frequency range and learning-induced theta-gamma interactions (Bach et al., 1995; Alberi et al., 2000; Barcomb et al., 2016). However, the role of hippocampal CaMKII in ORM destabilization and reconsolidation and its potential influence on oscillatory dynamics during these processes have not been previously investigated.

Here we show that intra-CA1 micro-infusion of the specific CaMKII inhibitor autocamtide-2-related inhibitory peptide (AIP; Ishida et al., 1995) does not affect ORM expression or persistence. However, it abolishes the increase in hPAC induced by ORM reactivation in the presence of a novel object and the amnesia triggered by post-reactivation intra-CA1 administration of the protein synthesis blocker anisomycin (ANI). These results suggest that hippocampal CaMKII regulates novelty detection-induced ORM destabilization through hPAC modulation but is not required for ORM recall or reconsolidation.

## Materials and methods

### Subjects

Three-months-old male Wistar rats were kept at 23°C with *ad libitum* access to food and water, following a 12 h light/dark cycle (lights on at 6:00 A.M.). Experiments were carried out during the light phase of the cycle following the National Institutes of Health’s Guide for Care and Use of Laboratory Animals and the ARRIVE guidelines. All procedures received approval from the local ethics committee.

### Stereotaxic surgery

Rats were anesthetized with a combination of ketamine (80 mg/kg) and xylazine (10 mg/kg) before being implanted with 22-gauge stainless steel guides (AP −4.2; LL, ±3.0; DV, −3.0) and/or recording electrodes (50 μm diameter PFA coated tungsten micro-wires). The cannula-electrode array consisted of two pairs of two electrodes. The electrodes in each pair were separated by 250 μm. Both pairs were placed parallel to the cannula, one on the left and the other on the right. The distance between the electrode pairs was approximately 1.5 mm, which accounted for the 0.7 mm width of the cannula, plus a 350 μm separation between each pair of electrodes and the cannula. The implant was inserted perpendicular to the brain surface, with its center located at coordinates AP −4.2; LL +3.0; DV −3.5 mm) targeting the CA1 region of the dorsal hippocampus using an automated stereotaxic system. Screws implanted in the skull were used as ground. Meloxicam (0.2 mg/kg) was administered as an analgesic at the end of the surgery. Animals were allowed to recover from surgery for at least 7 days before any other procedures. The stereotaxic coordinates were based on the work of Paxinos and Watson (Paxinos and Watson, 2007).

### Novel object recognition task (NOR)

Object recognition memory was assessed using the two-items version of the NOR task, a long-term ORM learning paradigm that leverages rats’ innate predilection for novelty. The procedures were conducted in open-field arenas made of uniformly painted gray plywood (60 × 60 × 60 cm), devoid of spatial or contextual cues, and placed in a dimly lit room kept at 23°C–24°C. Initially, animals were handled and habituated to the arena for 20 min per day over 4 days, with no objects present. One day after the final habituation session, rats were exposed to two different but behaviorally equivalent novel objects (referred to as objects A and B) for 3 or 5 min in the training arena (Training session - TR). The objects, made of metal, glass, or ceramic were chosen to ensure no innate preference (Rossato et al., 2019). Exploration events were defined as episodes lasting at least 0.5 s, during which rats sniffed or touched the objects with their snouts or paws. Behaviors such as sitting on or turning around the objects did not count as exploration. ORM reactivation was achieved by re-exposing rats to one of the objects presented during training (object A) along with a novel object (object C) for 5 min (Reactivation session - RA). Control experiments involved re-exposing animals to the same two objects used in the training session. Memory retention for object A memory was evaluated either 1 or 7 days post-reactivation by re-exposing rats to object A alongside a novel object (object D) during the Test session (TT). One hour before each experimental session, rats were transported from the vivarium to the experimental anteroom and then individually transferred to the experimental room in a transport cage. After each session, rats were returned to the experimental anteroom for an additional hour before being taken back to the vivarium. The open-field arenas and objects were cleaned with 50% ethanol prior to each trial. Video cameras mounted above the arenas tracked and recorded (30 frames/s) rats’ position and behaviors, which were analyzed using the ObjectScan system (CleverSys, RRID:SCR_017141). The discrimination index (DI) was calculated as (Time exploring novel object - Time exploring familiar object)/Total object exploration time. DI scores range from −1 and +1, with positive scores indicating a preference for the novel object, while scores close to zero suggest no discrimination.

### Drugs and microinjection procedures

Myristoylated AIP (Ishida and Fujisawa, 1995; Gailly, 1998; Tinsley et al., 2009; Bodhinathan et al., 2010; Mockett et al., 2011) was obtained from FastBio. Anisomycin (ANI) and clasto-lactacystin β-lactone (LAC) were sourced from Merck-Sigma Aldrich. Upon arrival, the drugs and peptides were resuspended, aliquoted, and stored at −20°C. Stock aliquots were diluted to the working concentration in sterile saline (VEH) immediately before the experiments. Microinjections (1 μl/side) were performed in a cleanroom next to the experimental room. Infusers were fitted to the guide cannulas and connected to Hamilton syringes using Tygon tubing. The flow rate was set at 0.5 μl/min and controlled by micro-infusion pumps. After the microinjections were completed, infusers were left in place for 1 min to minimize back-flow. Cannula placement was verified postmortem 1 day after the last behavioral test. Only data from animals with correctly placed implants were included in the analyses.

### *In vivo* electrophysiology

Local field potentials (LFP) were recorded using a Cerebus System (Blackrock Microsystems). The signals were amplified, digitized, filtered at cut-off frequencies of 0.3-Hz and 250-Hz, and sampled at 1-kHz. A 60-Hz digital notch filter was used to cancel line noise. Baseline electrode impedance was measured at 1 kHz in PBS prior to implantation, with values ranging from 13 to 20 kΩ. Impedance was also measured 7–10 days after surgery, with values ranging from 25 to 40 kΩ. Offline analyses were performed using MATLAB-routines (RRID:SCR_001622). Electrodes presented similar LFP signals; the electrode with the highest theta/delta ratio was selected for analysis. Power spectra were computed using the Welch’s method (“pwelch” function, 3 s Hamming windows, 75% overlap). Theta (5–10 Hz), slow gamma (35–55 Hz), and fast gamma (65–100 Hz) power were computed by integrating the power spectral density within their respective frequency ranges using the “bandpower” function. To evaluate the effect of intra-CA1 VEH and AIP infusions on spontaneous hippocampal oscillatory activity in freely moving naive rats, power changes between pre- (baseline) and post-infusion time points were calculated using 5 min epochs. Power changes were calculated using the formula: (power at the post-infusion time point/power at baseline) × 100. Results were expressed as a percentage of baseline activity. Baseline signals were recorded while the rats remained in a familiar arena. Afterward, the headstage was disconnected from the implant, and the animals were briefly removed from the arena for drug or vehicle infusion. Immediately following the infusion, the rats were reconnected to the recording system and returned to the arena to record post-infusion neural activity. To analyze the effect of intra-CA1 VEH and AIP infusions on hippocampal oscillatory activity during ORM reactivation, animals were first trained in the ORM task. One day later, they received intra-CA1 infusions of either VEH or AIP. Twenty minute after infusion, they were connected to the recording system and returned to the training arena with two objects. One subgroup explored a familiar object (A) alongside a novel object (C), while the other subgroup explored two familiar objects (A and B). LFP windows during object A exploration were extracted, merged, and analyzed. Exploration events shorter than 0.5 s were excluded, while events separated by less than 0.5 s were combined. Hippocampal phase-amplitude coupling between theta and gamma bands was measured using the Tort modulation index (MI). To compute hPAC, theta phase and gamma amplitude were derived from the Hilbert transform of the filtered versions of each frequency band. Theta phases were binned into 20° intervals, and the mean gamma amplitude was calculated for each theta phase bin and normalized by the sum of amplitude values over all bins. Comodulograms were obtained by expressing the modulation index (MI) of several frequency band pairs (4 Hz bandwidths, 1 Hz steps for phase frequencies; 10 Hz bandwidths, 2 Hz steps for amplitude frequencies) in pseudo-color plots. Mean MI, defined as the average MI values in the (5–10 Hz) × (35–55 Hz) or (5–10 Hz) × (65–100 Hz) regions of the comodulograms, was used to express cross-frequency coupling strength. Gamma events were defined as intervals during which power exceeded 2.5 sd above the time-averaged power, with intervals showing power greater than 6 sd excluded. Events separated by less than 100 ms were merged. The theta phase at time points corresponding to the peak of each gamma event was extracted to calculate the circular mean, yielding a single-phase value associated with high-gamma amplitude occurrence. The first 20 s of object A exploration were analyzed for each animal.

### Experimental design and data analyses

Subjects were randomly assigned to experimental groups, and researcher were blinded to the treatment conditions of the animals. Analyses were conducted using GraphPad Prism 10 software (RRID:SCR_002798), with significance set at *p* < 0.05. Novel object recognition data were analyzed using one-sample *t*-test (theoretical mean = 0), unpaired Student’s *t*-test, or one-way/two-way ANOVA followed by Bonferroni’s multiple comparisons as proper. Electrophysiological data were analyzed using ANOVA, repeated-measures two-way ANOVA, or a mixed-effects model, also followed by Bonferroni’s multiple comparisons test, as appropriate.

## Results

**FIGURE 1:**
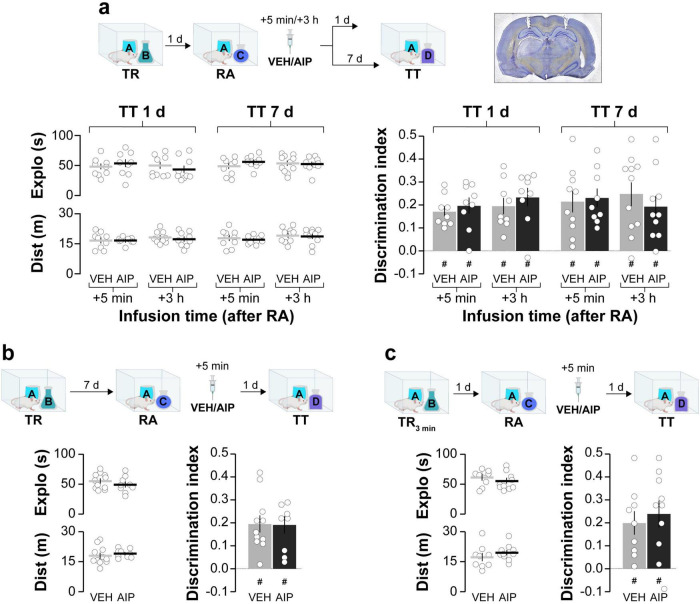
Post-reactivation inhibition of hippocampal CaMKII does not induce amnesia for ORM. **(a)** Adult male Wistar rats were trained in the NOR task using objects A and B (TR). One day after TR, they were re-exposed to familiar object A alongside novel object C to reactivate (RA) object A memory under conditions that promote reconsolidation. Five minute or 3 h after RA, rats received bilateral intra-dorsal CA1 infusions of either VEH or AIP. Memory retention was assessed 1 day or 7 days later in the presence of familiar object A and novel object D (TT). A photomicrograph displaying cannula placement tracks in the dorsal hippocampus is shown to the right of the schematics illustrating the experimental design. The left graphs display total exploration time and distance traveled, while the right graph shows the discrimination index during TT for both VEH and AIP-treated animals. **(b)**. In a similar setup, adult male Wistar rats trained in the NOR task using objects A and B (TR) were re-exposed to familiar object A alongside novel object C 7 days post-training to reactivate object A memory (RA). Five minute later, animals received bilateral intra-dorsal CA1 infusions of VEH or AIP. Memory retention was evaluated 1 day thereafter in the presence of familiar object A and novel object D (TT). The left graphs show total exploration time and distance traveled, while the right graph displays the discrimination index during TT for both treatment groups. **(c)** Another group of adult male Wistar rats trained in the NOR task using objects A and B for 3 min instead of 5 (TR_3min_) were also re-exposed to familiar object A alongside novel object C 1 day post-training (RA). Following a 5 min interval, rats received bilateral intra-dorsal CA1 infusions of VEH or AIP. Memory retention was evaluated 1 day later in the presence of familiar object A and novel object D (TT). The left graphs illustrate total exploration time and distance traveled, while the right graph indicates the discrimination index during TT for both VEH and AIP-treated animals. Data are presented as mean ± SEM; *n* = 9–11 animals per group. Dashed lines represent chance level. #*p* < 0.05 in one-sample Student’s *t*-test with theoretical mean = 0.

First, we analyzed the role of hippocampal CaMKII in ORM reconsolidation. We trained adult male Wistar rats in the novel object recognition task (NOR; Ennaceur and Delacour, 1988; Akirav and Maroun, 2006; ILL-Raga et al., 2013), an ORM-inducing learning paradigm based on rodents’ innate preference for novelty. During the training phase, the rats were exposed to two novel, different, but behaviorally equivalent objects, A and B, in a familiar open-field arena for 5 min. One day after training, the animals were re-exposed to object A alongside a novel object, C, in the training arena for another 5 min to reactivate the memory of object A, induce its destabilization, and elicit hippocampus-dependent reconsolidation. Five minutes or 3 h after reactivation, the animals received bilateral intra-dorsal CA1 infusions of either vehicle (VEH; saline) or the cell-permeable myristoylated form of the specific substrate-competitive CaMKII inhibitor AIP (5 nmol/side). Object A memory retention was assessed 1 day or 7 days later by re-exposing the animals to object A together with a novel object, D, for 5 min. AIP did not alter total exploration time or total distance traveled, and regardless of treatment, injection time, or the interval between reactivation and testing, all groups preferentially explored object D and successfully discriminated it from object A during the retention test session (Figure 1a). Memory age and strength at the time of reactivation may influence reconsolidation (Nader and Einarsson, 2010). Therefore, we examined whether increasing the training-reactivation time interval or shortening the training session could enable the amnesic effect of AIP. We found that post-reactivation intra-CA1 administration of AIP had no effect on ORM retention, even when the reactivation session was conducted 7 days instead of 1 day post-training (Figure 1b) or when the training session lasted 3 min instead of 5 min (Figure 1c). These results confirm that hippocampal CaMKII inhibition does not cause reactivation-dependent amnesia for ORM. Exploration and discrimination data from both the training and reactivation sessions are shown in Table 1.

**TABLE 1:** Exploration time and discrimination index (DI) during NOR training (TR) and reactivation (RA) sessions for animals in Figures 1–3.

			TR				RA			
Figures	Time	Treatment	Exploration (s)		DI		Exploration (s)		DI	
			+5 min	+3 h	+5 min	+3 h	+5 min	+3 h	+5 min	+3 h
1a	1 day	VEH	64.97 ± 3.8	61.02 ± 6.1	0.020 ± 0.02	−0.009 ± 0.03	59.16 ± 4.5	60.10 ± 3.5	0.22 ± 0.04	0.19 ± 0.03
		AIP	62.94 ± 3.6	58.23 ± 5.4	−0.004 ± 0.03	−0.007 ± 0.02	53.81 ± 5.8	55.03 ± 5.8	0.25 ± 0.04	0.23 ± 0.03
	7 days	VEH	56.90 ± 5.3	60.56 ± 3.7	0.002 ± 0.03	−0.015 ± 0.02	60.14 ± 3.8	57.47 ± 4.1	0.21 ± 0.04	0.21 ± 0.04
		AIP	55.85 ± 3.3	56.72 ± 4.2	0.008 ± 0.03	−0.015 ± 0.03	57.57 ± 6.4	58.01 ± 5.1	0.21 ± 0.06	0.23 ± 0.07
1b	1 day	VEH	63.05 ± 5.5		0.016 ± 0.03		54.24 ± 4.5		0.23 ± 0.04	
		AIP	57.58 ± 5.7		−0.034 ± 0.03		55.26 ± 4.7		0.26 ± 0.03	
1c	1 day	VEH	56.46 ± 5.2		−0.027 ± 0.04		60.32 ± 4.1		0.20 ± 0.06	
		AIP	57.46 ± 4.5		0.001 ± 0.03		58.91 ± 3.9		0.22 ± 0.04	
2	1 day	VEH	55.26 ± 3.5		0.020 ± 0.02		–		–	
		AIP	48.46 ± 2.3		0.002 ± 0.02		–		–	
3a	1 day	VEH + VEH	55.52 ± 3.7		0.03 ± 0.03		56.08 ± 4.4		0.22 ± 0.03	
		AIP + VEH	55.32 ± 3.0		−0.01 ± 0.02		53.88 ± 3.7		0.23 ± 0.04	
		VEH + ANI	50.24 ± 3.2		−0.02 ± 0.02		58.98 ± 4.4		0.20 ± 0.02	
		AIP + ANI	50.02 ± 3.2		−0.01 ± 0.02		51.82 ± 2.5		0.21 ± 0.03	
3b	1 day	VEH	54.81 ± 3.3		0.01 ± 0.03		51.20 ± 3.9		0.21 ± 0.04	
		AIP	55.18 ± 6.2		0.05 ± 0.03		53.45 ± 6.0		0.24 ± 0.05	
		ANI	52.46 ± 3.8		0.05 ± 0.04		56.82 ± 4.0		0.22 ± 0.04	
		AIP + ANI	58.58 ± 3.9		0.01 ± 0.03		55.31 ± 3.1		0.22 ± 0.03	

Data are presented as mean ± SEM.

**FIGURE 2:**
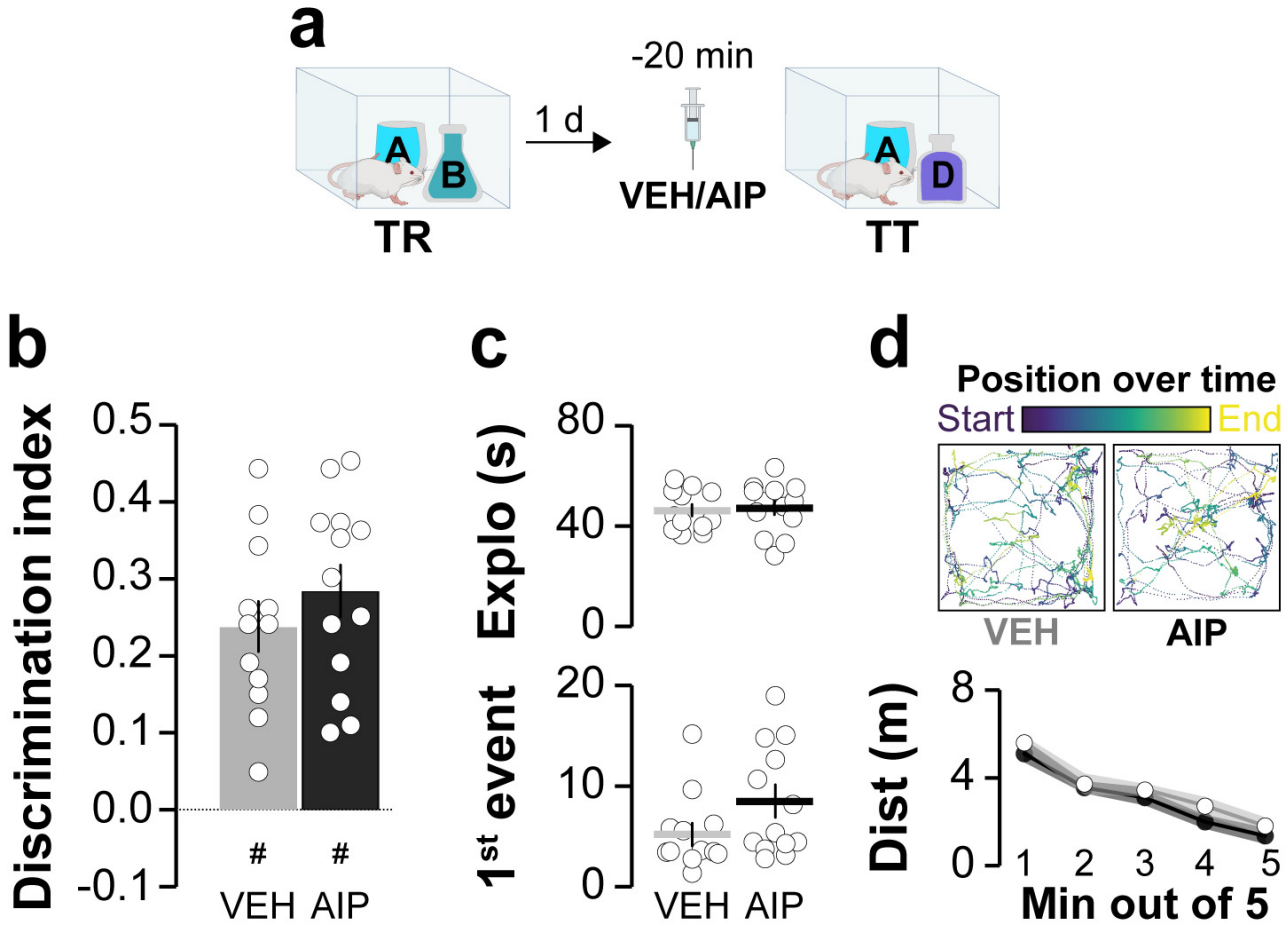
Inhibiting hippocampal CaMKII does not affect ORM recall. **(a)** Adult male Wistar rats trained in the NOR task with objects A and B (TR) received bilateral intra-dorsal CA1 infusions of either VEH or AIP 1 day post-training. ORM retention was evaluated 20 min later in the presence of familiar object A and novel object D (TT). **(b)** Discrimination index, (**c**, Top panel) total exploration time, and (**c**, Bottom panel) latency to the first exploration event during TT. (**d**, Top panel) Traces show the trajectory and position during TT for representative VEH- and AIP-treated rats. (**d**, Bottom panel) Distance traveled during TT. Data are presented as mean ± SEM; *n* = 13 animals per group. Dashed line indicates chance level. #*p* < 0.05 in one-sample Student’s *t*-test with theoretical mean = 0.

**FIGURE 3:**
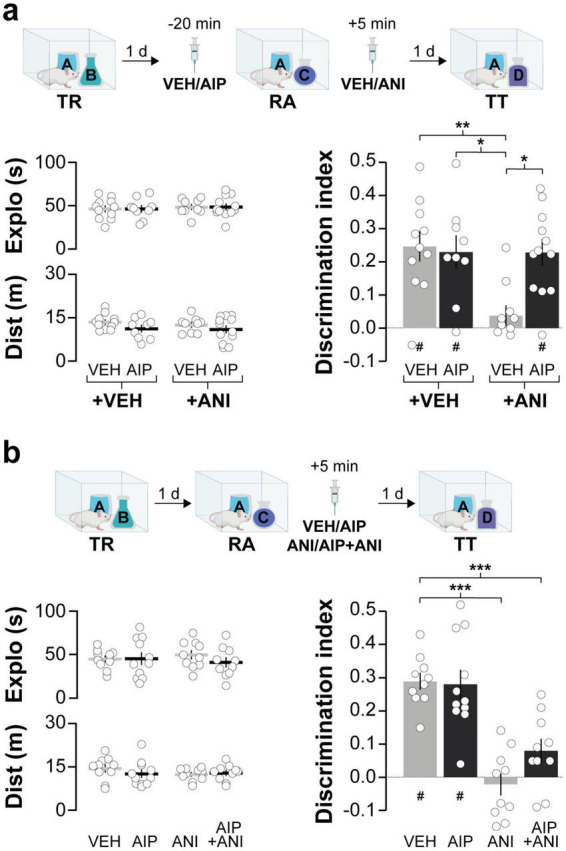
Inhibiting hippocampal CaMKII prior to ORM reactivation prevents the amnesic effect of the protein synthesis blocker anisomycin. **(a)** Adult male Wistar rats trained in the NOR task using objects A and B (TR) underwent ORM reactivation (RA) 1 day post-TR, in the presence of familiar object A and novel object C. Twenty minutes before RA, the animals received bilateral intra-dorsal CA1 infusions of either VEH or AIP. Five minutes later, they were administered either VEH or ANI in CA1. Memory retention was evaluated 1 day later in the presence of familiar object A and novel object D (TT). The left graphs illustrate total exploration time and distance traveled, while the right graph indicates the discrimination index during TT. **(b)** In a separate experiment, adult male Wistar rats trained in the NOR task with objects A and B (TR) were subjected to RA in the presence of familiar object A and novel object C, 1 day post-TR. Five minute later, they received bilateral intra-dorsal CA1 infusions of VEH, AIP, ANI, or a combination of AIP and ANI, followed by an ORM retention test 24 h later (TT). The left graphs illustrate total exploration time and distance traveled, while the right graph indicates the discrimination index during TT. Data are presented as mean ± SEM; *n* = 9–12 animals per group. Dashed lines indicate chance level. #*p* < 0.05 in one-sample Student’s *t*-test with theoretical mean = 0; *p < 0.05, **p < 0.01 and ***p < 0.001 in Bonferroni test after one- or two-way ANOVA.

Next, we investigated the involvement of hippocampal CaMKII in ORM destabilization. Earlier research has shown that blocking protein synthesis in dorsal CA1 shortly after reactivation in the presence of a novel object prevents ORM reconsolidation and leads to amnesia (Rossato et al., 2007). So, we hypothesized that if hippocampal CaMKII is needed for ORM destabilization, then intra-CA1 administration of AIP prior to memory reactivation should mitigate the amnestic effect of inhibiting protein synthesis in the hippocampus. This reasoning aligns with approaches used before to study recall-induced destabilization in other types of memory (Milton et al., 2013; Lee and Flavell, 2014). Before testing our hypothesis regarding hippocampal CaMKII and ORM destabilization, we established that AIP does not affect ORM recall when infused into dorsal CA1 20 min prior to a retention test session conducted 1 day post-training (Figure 2). We then trained animals in the NOR task using objects A and B as stimuli and, 20 min before a reactivation session conducted 1 day post-training in the presence of familiar object A and novel object C, we injected AIP or VEH into dorsal CA1. Five minutes after ORM reactivation, animals received either VEH or the protein synthesis inhibitor anisomycin (ANI; 160 μg/side) in dorsal CA1. Object A memory retention was assessed 1 day later in the presence of novel object D. Animals that received VEH both before and after reactivation successfully discriminated object A from object D during the retention test session. However, those that received VEH before reactivation and ANI afterward showed amnesia, confirming that ORM reconsolidation requires protein synthesis in dorsal CA1. In support of the notion that hippocampal CaMKII plays a critical role in ORM destabilization, pre-reactivation intra-dorsal CA1 administration of AIP prevented the amnesia caused by ANI (Figure 3a; F(1,38) = 4.418, *p* = 0.0422 for Pre-RA Treatment; F(1,38) = 6.451, *p* = 0.0153 for Post-RA Treatment and F(1,38) = 6.339, *p* = 0.0161 for Interaction; t(38) = 3.593, *p* < 0.01 for VEH + VEH vs. VEH + ANI; t(38) = 3.136, *p* < 0.05 for AIP + VEH vs. VEH + ANI and t(38) = 3.349, *p* < 0.05 for AIP + ANI vs. VEH + ANI in Bonferroni’s multiple comparisons test after two-way ANOVA). However, AIP did not prevent the amnestic effect of ANI when both were co-infused into dorsal CA1 5-min post-reactivation (Figure 3b; F(3,37) = 19.66, *p* < 0.001; t(37) = 6.297, *p* < 0.001 for VEH + VEH vs. VEH + ANI; t(37) = 4.225, *p* < 0.001 for VEH + VEH vs. AIP + ANI; t(37) = 6.258, *p* < 0.001 for VEH + ANI vs. AIP + VEH and t(37) = 4.137, *p* < 0.01 for AIP + VEH vs. AIP + ANI in Bonferroni’s multiple comparisons test after one-way ANOVA).

**FIGURE 4:**
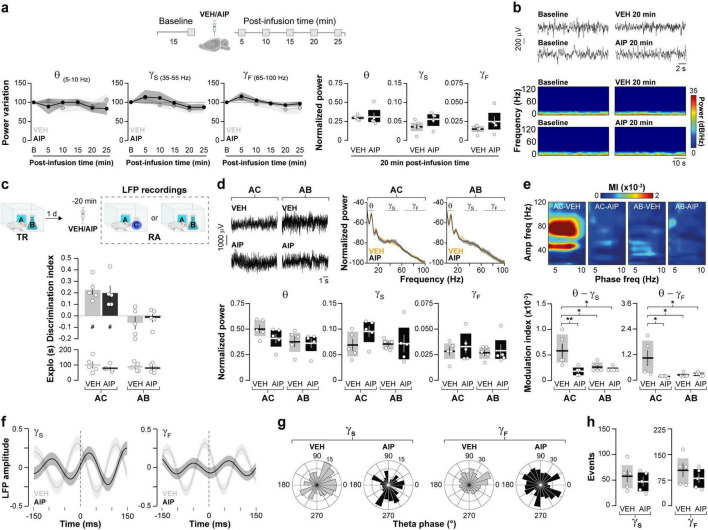
Inhibiting hippocampal CaMKII disrupts hPAC induced by ORM destabilization. **(a,b)** Naïve adult male Wistar rats implanted with cannulas and electrodes targeting the dorsal CA1 region were used to record hippocampal LFP signals before (Baseline - B) and at various times (5–25 min) after intra- dorsal CA1 infusions of either VEH or AIP. **(a)** The power variation (expressed as a percentage of baseline) at each time point, along with the normalized theta (5–10Hz), slow gamma (35–55 Hz), and fast gamma (65–100 Hz) power during the 20-min interval is shown. **(b)** Representative raw LFP traces and spectrograms for baseline and 20 min post-infusion time points; *n* = 5 animals per group. **(c)** Adult male Wistar rats, also implanted with cannulas and electrodes targeting the dorsal CA1 region, were trained (TR) in the NOR task using objects A and B. One day after TR, they received bilateral intra-dorsal CA1 infusions of either VEH or AIP. Twenty minute later, a memory reactivation session (RA) for object A was conducted, during which hippocampal LFPs were recorded and data corresponding to object A exploration were analyzed. RA was performed under conditions that either promoted object A memory destabilization (in the presence of a novel object C) or impeded it (in the presence of familiar object B). Importantly, AIP did not affect object exploration or the recall of memories for objects A and B during RA. (**d**, Top panel) Representative raw LFP traces and power spectrum density plots. (**d**, Bottom panel) Shows normalized theta, slow gamma, and fast gamma power. (**e**, Top panel) Presents representative phase-amplitude comodulograms (MI, modulation index). (**e**, Bottom panel) Illustrates, theta-slow gamma and theta-fast gamma modulation index. **(f)** Shows averaged LFP signals (z-score) triggered by the peak of gamma events. **(g)** Features circular histograms depicting the distribution of slow and fast gamma events within the theta cycle. **(h)** Displays the total number of gamma events. The first 20 s of object A exploration were analyzed for each animal. Data are presented as mean ± SEM; *n* = 5 animals per group. #*p* < 0.05 in one-sample *t*-test with theoretical mean = 0; *p < 0.05 and **p < 0.01 in Bonferroni test following two-way ANOVA.

**FIGURE 5:**
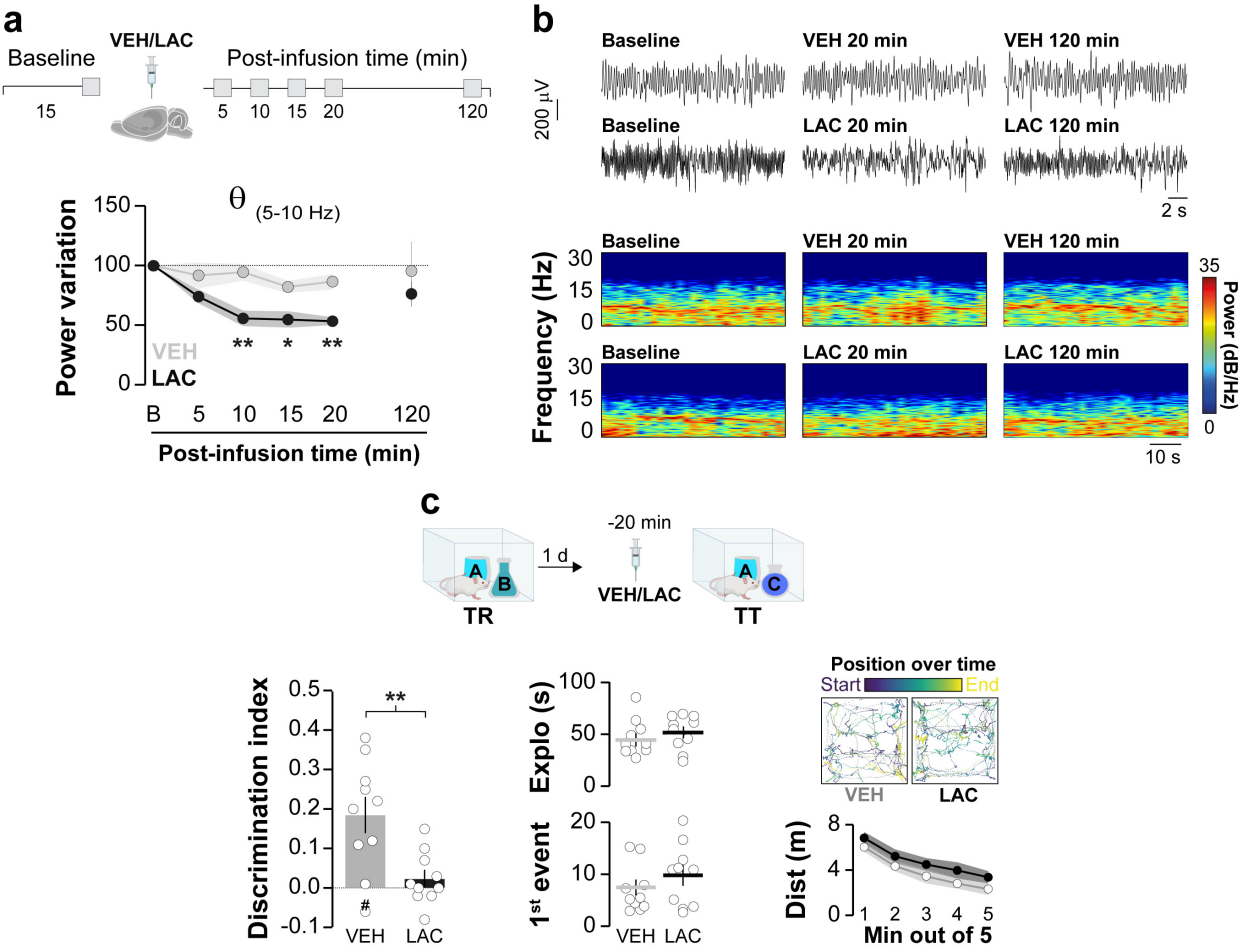
Intra-hippocampal administration of clasto-lactacystin β-lactone (LAC) disrupts spontaneous theta activity and hinders ORM recall. **(a,b)** Naïve adult male Wistar rats, fitted with cannulas and electrodes targeting the dorsal CA1 region, were used to record hippocampal LFP signals before (Baseline - B) and at various time points (5–120 min) following intra-dorsal CA1 infusions of either VEH or LAC. **(a)** The normalized power of theta frequencies (5–10Hz) is presented. **(b)** Representative raw LFP traces and spectrograms for baseline, 20 min and 120 min post-infusion time points are shown; *n* = 5 animals per group. **(c)** Adult male Wistar rats implanted with cannulas targeting the dorsal CA1 region were trained in the NOR task using objects A and B (TR). One day after TR, they received bilateral intra-dorsal CA1 infusions of either VEH or LAC. Twenty minutes later, the rats underwent a retention test session (TT) in the presence of familiar object A and novel object C. (Left panel) Displays the discrimination index; (Center panel) Shows total exploration time and latency to the first exploration event; (Right panel) Illustrates trajectory, position, and distance traveled during TT for both VEH- and LAC-treated rats. Data are presented as mean ± SEM; *n* = 10 animals per group. The dashed line indicates chance level. #*p* < 0.05 in one-sample Student’s *t*-test with theoretical mean = 0; *p < 0.05 and **p < 0.01 in Bonferroni test after mixed-effect analysis or Student’s *t*-test, as appropriate.

Synchronous neuronal electrical activity results in rhythmic local field potential (LFP) fluctuations known as neural oscillations (Buzsáki, 2012). In the brain, these oscillations are observed at various levels of organization and are thought to be crucial for sensory-cognitive processes (Klimesch et al., 2010; Singer, 2018; Ghiani et al., 2021). Specifically, hippocampal neural oscillations in the theta and gamma frequency bands are linked to different memory processes (Jensen and Colgin, 2007). Novelty-induced ORM destabilization triggers theta-gamma cross-frequency coupling in dorsal CA1 (hPAC), while experimental manipulations that artificially induce hPAC during recall can render destabilization-resistant ORMs susceptible to reconsolidation blockers (Gonzalez et al., 2021b). Therefore, we studied the effect of intra-dorsal CA1 AIP administration on hPAC during ORM reactivation. Before conducting this experiment, we confirmed that injections of either VEH or AIP into the CA1 region of the dorsal hippocampus did not affect spontaneous neural oscillatory activity in freely moving naïve adult male rats (Figures 4a, b; Theta: F(1,8) = 0.0657, *p* = 0.8041 for treatment; F(2.045,16.37) = 0.3963, *p* = 0.6837 for time; F(4,32) = 1.383, *p* = 0.2618 for interaction, ε = 0.5117; Slow Gamma: F(1,8) = 0.4552, *p* = 0.5189 for treatment; F(1.524,12.19) = 2.689, *p* = 0.1167 for time; F(4,32) = 0.2451, *p* = 0.9105 for interaction, ε = 0.3810; Fast Gamma: F(1,8) = 0.7262, *p* = 0.4189 for treatment; F(1.928,15.43) = 3.681, *p* = 0.0507 for time; F(4,32) = 0.1727, *p* = 0.9508 for interaction, ε = 0.4821 in mixed-effects model analysis with Geisser-Greenhouse’s sphericity correction). Importantly, theta, slow gamma and fast gamma power during the 20–25 min post-infusion bin did not differ between VEH and AIP groups (Theta: t(8) = 0.241, *p* = 0.815, Slow Gamma: t(8) = 1.85, *p* = 0.101, Fast Gamma: t(8) = 1.41, *p* = 0.196, unpaired *t*-test). Then, we implanted rats with cannulas and electrodes in dorsal CA1 and trained them in the NOR task using novel objects A and B as stimuli. One day after training, the animals received intra-dorsal CA1 injections of either VEH or AIP and 20 min later they underwent an ORM reactivation session with familiar object A and novel object C to induce object A memory destabilization. To differentiate destabilization-specific mechanisms from those merely associated with ORM recall, we subjected a different group of NOR-trained animals to ORM reactivation with familiar objects A and B, which triggers object A memory recall but does not induce its destabilization (Myskiw et al., 2008; Radiske et al., 2017). LFP were recorded throughout the reactivation session, and data from time windows corresponding to object A exploration epochs were extracted, merged, and analyzed. As expected, animals exposed to familiar object A and novel object C preferentially explored the latter during the reactivation session, while animals exposed to familiar objects A and B spent equal time exploring both (Figure 4c, F(1,16) = 0.02042, *p* = 0.8882 for Treatment; F(1,16) = 27.10, *p* < 0.0001 for RA; F(1,16) = 0.5105, *p* = 0.4852 for interaction; *p* = 0.0042 for VEH/AC vs. VEH/AB, *p* = 0.0150 for VEH/AC vs. AIP/AB, *p* = 0.0098 for VEH/AB vs. AIP/AC, *p* = 0.0352 for AIP/AC vs. AIP/AB in Bonferroni’s multiple comparisons test after two-way ANOVA). Total exploration time (Figure 4c; F(1,16) = 0.9927, *p* = 0.3339 for Treatment; F(1,16) = 0.1589, *p* = 0.6955 for RA; F(1,16) = 0.1959, *p* = 0.6639 for interaction in two-way ANOVA), locomotion (VEH/AB: M = 29.69, SEM = 2.99; AIP/AB: M = 23.93, SEM = 2.39; VEH/AC: M = 25.26, SEM = 2.54; AIP/AC: M = 26.40, SEM = 3; F(1,16) = 0.7109, *p* = 0.4116 for Treatment; F(1,16) = 0.1280, *p* = 0.7252 for RA; F(1,16) = 1.580, *p* = 0.2268 for interaction in two-way ANOVA), and theta, slow gamma, and fast gamma power did not differ between groups, regardless of treatment (Figure 4d; Theta: F(1,16) = 1.382, *p* = 0.2569 for Treatment; F(1,16) = 3.575, *p* = 0.0769 for RA; F(1,16) = 0.7052, *p* = 0.4134 for interaction; Slow Gamma: F(1,16) = 1.375, *p* = 0.2582 for Treatment; F(1,16) = 0.8661, *p* = 0.3659 for RA; F(1,16) = 1.204, *p* = 0.2888; Fast Gamma: F(1,16) = 0.0578, *p* = 0.8130 for Treatment; F(1,16) = 0.6775, *p* = 0.4225 for RA; F(1,16) = 0.2974, *p* = 0.5930 for interaction in two-way ANOVA). Confirming earlier findings (Gonzalez et al., 2021a,2021b), control animals exposed to familiar object A and novel object C, but not those exposed to familiar objects A and B, displayed strong hPAC during object A exploration. Conversely, animals that received AIP before reactivation in the presence of objects A and C showed reduced hPAC (Figure 4e; Theta-Slow gamma: F(1,16) = 8.666, *p* = 0.0095 for Treatment, F(1,16) = 3.677, *p* = 0.0732 for RA, F(1,16) = 6.040, *p* = 0.0258 for interaction; *p* = 0.0418 for VEH/AC vs. VEH/AB, *p* = 0091 for VEH/AC vs. AIP/AC, *p* = 0.0203 for VEH/AC vs. AIP/AB. Theta-Fast gamma: F(1,16) = 5.337, *p* = 0.0346 for Treatment, F(1,16) = 3.889, *p* = 0.0661 for RA, F(1,16) = 6.429, *p* = 0.0220 for interaction; *p* = 0.0344 for VEH/AC vs. VEH/AB, *p* = 0208 for VEH/AC vs. AIP/AC, *p* = 0.0480 for VEH/AC vs. AIP/AB, in Bonferroni’s multiple comparisons test after two-way ANOVA). AIP administration also altered the distribution of gamma events over the theta cycle (Figures 4f, g; Slow gamma: Z = 6.11, *p* = 0.001 for VEH. Fast gamma: Z = 10.88, *p* < 0.0001 for VEH), showing that hippocampal CaMKII activity during reactivation is necessary for ORM destabilization-induced hPAC. Notably, the total number of gamma events was similar between groups (Figure 4h; Slow Gamma events: t(8) = 0.776, *p* = 0.460, Fast Gamma events: t(8) = 1.004, *p* = 0.345, unpaired *t*-test). While we cannot rule out the possibility that sniffing, whisking, or locomotion may influence the oscillatory activity analyzed, animals across all groups exhibited similar behavior. Strong theta-gamma coupling is typically associated with high-velocity bins (>35 cm/s), whereas the data analyzed in our study focus on moments of active exploration (i.e., when animals engage with the object at reduced locomotion speeds of ∼5–15 cm/s). Although whisking and sniffing were not specifically measured, these behaviors were observed in all animals during the 20 s of object A exploration analyzed. Earlier reports suggest that synaptic protein degradation drives reactivation-induced memory destabilization (Lee et al., 2008), and it is known that active CaMKII can recruit proteasomes to dendritic spines (Bingol et al., 2010). Moreover, inhibiting amygdalar CaMKII hinders the destabilization of contextual fear conditioning (CFC) memory by blocking proteasome activity, which increases in a CaMKII-dependent manner 90 min after CFC recall (Jarome et al., 2011, 2016). Interestingly, calcium influx through T-type channels, which regulates neuronal excitability (Aguado et al., 2016), theta activity (Arshaad et al., 2021), hPAC (Joksimovic et al., 2023), bidirectional plasticity (Leresche and Lambert, 2017), and memory recall (Chen et al., 2012; Gangarossa et al., 2014) also activates CaMKII (Pasek et al., 2015; Asmara et al., 2017) and controls CaMKII-dependent proteasome up-regulation in hippocampal neurons (Xu et al., 2021). This suggest that CaMKII-dependent hPAC may modulate proteolytic activity during the initial stages of the ORM destabilization and reconsolidation process. Indeed, theta-burst stimulation of the fimbria fornix, which induces artificial hPAC and destabilizes reconsolidation-resistant ORM (Gonzalez et al., 2021b), differentially modulates proteasome activity during early and late hippocampal LTP (Santos et al., 2015). However, we found that intra-dorsal CA1 administration of clasto-lactacystin β-lactone (LAC, 32 ng/side), a cell-permeable and irreversible proteasome inhibitor commonly used to assess the role of multi-subunit proteases in memory persistence (Lee et al., 2008; Felsenberg et al., 2014; Reis et al., 2013; Troyner and Bertoglio, 2020) resulted in a significant yet reversible decrease in hippocampal theta power in naïve rats (Figures 5a, b; F(2.474,19.79) = 16.85, *p* = 0.0001 for Time; F(1,8) = 12.56, *p* = 0.0076 for Treatment and F(4,32) = 5.981, *p* = 0.001 for interaction; VEH vs. LAC: 10 min t(7.925) = 4.039, *p* = 0.0038, 15 min t(7.288) = 3.23, *p* = 0.0137 and 20 min t(5.859) = 4.399, *p* = 0.0048 in Bonferroni’s multiple comparisons test after a mixed-effects model analysis with Geisser-Greenhouse’s sphericity correction) and impaired ORM recall (Figure 5c; t(18) = 3.268, *p* = 0.0043 in a Student’s *t*-test) without affecting exploration or locomotion, which prevented us from studying the potential interaction between hippocampal CaMKII, hPAC, and proteasome activity during ORM destabilization.

## Discussion

Our data confirm the involvement of the hippocampus in the ORM destabilization/reconsolidation cycle and reveal that hippocampal CaMKII inhibition does not induce reactivation-dependent amnesia or impair recall but prevents ORM destabilization by impeding hPAC. These results align with findings showing that intra-perirhinal cortex injection of the Ca2+/CaM-dependent kinases blocker KN-93 inhibits contextual novelty-induced destabilization of hippocampus-independent object memory (Winters et al., 2004; Wideman et al., 2023). They are also consistent with studies showing that post-reactivation CaMKII inhibition does not affect CFC but cancels the amnesic effect of intra-amygdala ANI administration by hindering memory destabilization via proteasome inhibition (Jarome et al., 2011). Indeed, several studies suggest that protein degradation acts upstream of protein synthesis during memory reconsolidation (Lee et al., 2008; Park and Kaang, 2019) and, notably, amygdalar proteasome activity increases in a CaMKII-dependent manner 90 min after CFC recall (Jarome et al., 2016). However, unlike CFC, ORM destabilization requires a rapid upstream mechanism that operates during reactivation, rather than afterward, to rapidly reflect changes in synaptic weight driven by information resulting from the immediate comparison of contiguous objects. In this context, hPAC coordinates neuronal activity at a timescale crucial for memory processing (Skaggs et al., 1996; Lisman et al., 2005) and is thought to temporally organize episodic representations while interweaving them with contextual information that reaches the hippocampus from various brain regions via the rhinal cortex (Burwell and Amaral, 1998; Remondes and Schuman, 2004; Nyhus and Curran, 2010; Bilash et al., 2023). In any case, it is essential to recognize that ORM and CFC are fundamentally distinct memory types, both biochemically and behaviorally. Unlike CFC (Schuette et al., 2016), ORM maintenance does not depend on hippocampal PKMζ, though this kinase is necessary for ORM reconsolidation (Rossato et al., 2019) which, unlike CFC reconsolidation (Lee et al., 2004), is driven by hippocampal BDNF (Radiske et al., 2017) in addition to Zif268 signaling (Gonzalez et al., 2019). Moreover, ORM destabilization is not triggered by the absence of expected stimuli, as is CFC, but rather by novelty perception and comparison with familiar stimuli during recall (Lima et al., 2009; Rossato et al., 2015). Notably, ORM is an inextinguishable declarative memory, whereas CFC is a non-declarative memory prone to extinction. While CFC reconsolidation and extinction can be dissociated (Lee, 2008), reconsolidation occurs only if the US is re-presented within the destabilization time window initiated by non-reinforced recall (Tay et al., 2019). This process may still engage competing mechanisms requiring CaMKII and proteasome activity (Kimura et al., 2008; Mao et al., 2008). Consequently, unlike ORM, the effect of peri-recall CaMKII inhibition on CFC cannot be unequivocally attributed solely to memory reconsolidation modulation (Vigil et al., 2017). Yet, it is noteworthy that intra-PRh administration of LAC impedes reactivation-induced destabilization of hippocampus-independent object memory regardless of the introduction of novelty during the reactivation session, albeit in the absence of increased PRh proteasome activity (Stiver et al., 2017). Additionally, earlier studies reported that intra-CA1 injection of LAC prevents novelty-induced ORM destabilization (Furini et al., 2015), although this occurred at doses between 2,000 and 4,000 times higher than typically used in such experiments (Reis et al., 2013; Cullen et al., 2017; Bernabo et al., 2021). This suggest that the observed results may not stem from inhibition of the hippocampal proteasome but rather from non-specific pharmacological interactions or behavioral side effects (Ostrowska et al., 2000; Papa and Rockwell, 2008). In fact, we were unable to confirm the potential role of the hippocampal proteasome on ORM destabilization because, when used at standard dosages, LAC impaired ORM recall and rapidly and significantly decreased hippocampal theta power in naive rats. This raises the intriguing possibility that some of the memory effects attributed to LAC may result from its non-specific actions on neuronal oscillations rather than the inhibition of proteolytic activity *per se*. Future experiments to identify CaMKII-dependent hPAC effectors during ORM destabilization should analyze their possible interplay with the proteasome, particularly that mediated by hippocampal GluN2B-NMDAR, which not only binds proteasomes recruited by CaMKII (Bingol et al., 2010; Ferreira et al., 2021) but also controls ORM destabilization and updating (Lisman et al., 2012). In this regard, it is important to highlight that ORM consolidation depends on hippocampal CaMKII activity (Tinsley et al., 2009; Rossato et al., 2025), and preventing ORM destabilization allows the novel object presented during reactivation (object C) to be encoded through consolidation rather than reconsolidation mechanisms (Gonzalez et al., 2021a). Consequently, this limitation prevented us from assessing the potential effects of hippocampal CaMKII inhibition on ORM updating. Since ORMs acquired via consolidation mechanisms are independent (Gonzalez et al., 2021b) and can be manipulated separately, evaluating the impact of CaMKII inhibition on ORM updating is far more complex than it appears. A comprehensive analysis would require treatments capable of specifically dissociating recall, destabilization, consolidation, and reconsolidation, an approach beyond the scope of this study.

Alzheimer’s disease (AD) is closely associated with a decline in ORM processing, which impairs the ability to acquire and integrate new declarative knowledge, often resulting in the loss of crucial personal memories (Caterini et al., 2002; Laatu et al., 2003). The accumulation of amyloid-β (Aβ) and the formation of neurofibrillary tangles from hyperphosphorylated tau (Knopman et al., 2021) are well-established pathological hallmarks of AD. Recent studies suggest that AD progression also involves early reorganization of hippocampal neuronal ensembles, a process associated with CaMKII dysfunction and dysregulated hippocampal oscillatory activity. Notably, disruptions in hPAC precede Aβ overproduction and deposition in various mouse models of AD (Goutagny et al., 2013; Mehak et al., 2022; Victorino et al., 2023), highlighting the close connection between AD-related toxicity and changes in neural network excitability and synchrony, potentially driven by CaMKII signaling dysfunction (Ghosh and Giese, 2015; Imfeld et al., 2013; Opazo et al., 2018; Brown et al., 2022; Brown and Bayer, 2024). In light of these findings, our results, which demonstrate that hippocampal CaMKII regulates hPAC and ORM destabilization, may have significant clinical implications for addressing declarative memory decline in AD. By emphasizing the role of CaMKII-regulated hPAC in reactivated ORM stability, our work suggest that targeting this pathway could be a promising therapeutic strategy, warranting further investigation.

Before concluding, it is important to note that this study was conducted using adult male rats. While we acknowledge the recent emphasis on incorporating both sexes in experimental designs, and recognize the biological rationale for doing so, we believe that limiting our study to males does not compromise the validity or generalizability of our findings. CaMKII plays a critical role in memory processing, and its function is highly conserved across species, from mollusks to mammals. Given this broad conservation, significant sexual dimorphism in CaMKII-mediated memory mechanisms is unlikely. Logistical, financial, and regulatory constraints, particularly related to breeding, housing, and maintenance, prevented the inclusion of females in the current study. This limitation was unintentional, and we plan to address both sexes in future investigations of hippocampal CaMKII’s role in ORM destabilization.

## Data Availability

The original contributions presented in this study are included in this article/supplementary material, further inquiries can be directed to the corresponding author.
